# Supplementary figures and images for: IBD risk locus rs1077773 enhances aryl hydrocarbon receptor activity and modulates immune cell function in vitro
Source: Inflamm Res. 2026 Apr 10;75(1):88. doi: 10.1007/s00011-026-02236-3 (PMC13068725; doi:10.1007/s00011-026-02236-3)

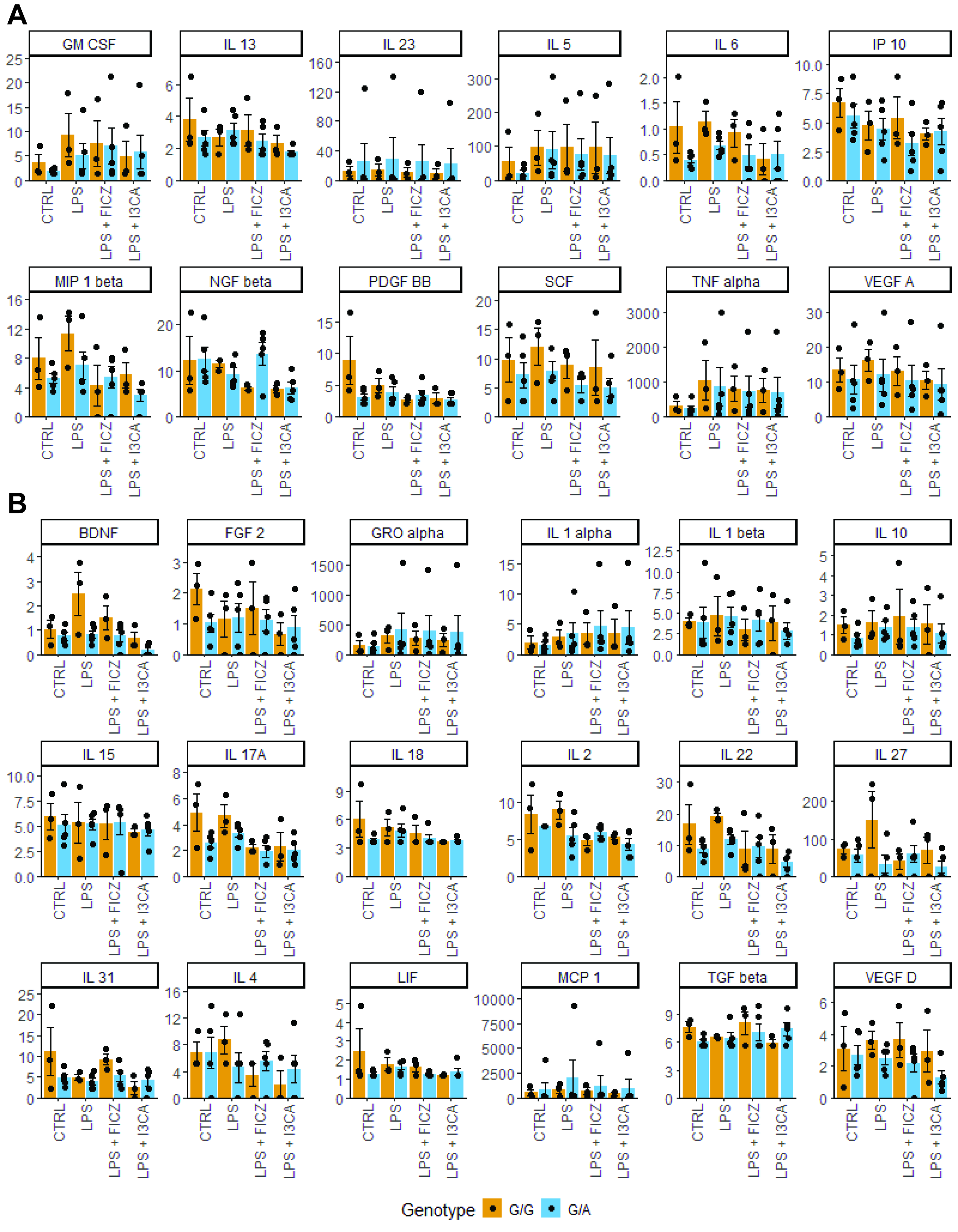

Supplement: Supplementary file 1 — Supplementary Material 1 [file 11_2026_2236_MOESM1_ESM.tif]
